# Enhancing Autonomous Vehicle Decision-Making at Intersections in Mixed-Autonomy Traffic: A Comparative Study Using an Explainable Classifier

**DOI:** 10.3390/s24123859

**Published:** 2024-06-14

**Authors:** Erika Ziraldo, Megan Emily Govers, Michele Oliver

**Affiliations:** School of Engineering, University of Guelph, Guelph, ON N1G 2W1, Canada; eziraldo@uoguelph.ca (E.Z.); mgovers@uoguelph.ca (M.E.G.)

**Keywords:** driver behaviour, machine learning, autonomous vehicles, driving simulator, vehicle-to-vehicle communication

## Abstract

The transition to fully autonomous roadways will include a long period of mixed-autonomy traffic. Mixed-autonomy roadways pose a challenge for autonomous vehicles (AVs) which use conservative driving behaviours to safely negotiate complex scenarios. This can lead to congestion and collisions with human drivers who are accustomed to more confident driving styles. In this work, an explainable multi-variate time series classifier, Time Series Forest (TSF), is compared to two state-of-the-art models in a priority-taking classification task. Responses to left-turning hazards at signalized and stop-sign-controlled intersections were collected using a full-vehicle driving simulator. The dataset was comprised of a combination of AV sensor-collected and V2V (vehicle-to-vehicle) transmitted features. Each scenario forced participants to either take (“go”) or yield (“no go”) priority at the intersection. TSF performed comparably for both the signalized and sign-controlled datasets, although all classifiers performed better on the signalized dataset. The inclusion of V2V data led to a slight increase in accuracy for all models and a substantial increase in the true positive rate of the stop-sign-controlled models. Additionally, incorporating the V2V data resulted in fewer chosen features, thereby decreasing the model complexity while maintaining accuracy. Including the selected features in an AV planning model is hypothesized to reduce the need for conservative AV driving behaviour without increasing the risk of collision.

## 1. Introduction

While autonomous vehicles (AVs) promise a large reduction in the estimated 90% of collisions with human factor causes [1], this milestone is not expected to be reached until at least 2050 [2]. The transition to adoption of full autonomy will include a long period of mixed-autonomy traffic. During this period, a changing mixture of vehicles with autonomy levels ranging from conventional to fully automated will be operating on the same roadways. The mixed-autonomy roadway creates a particular challenge for AVs which struggle to recognize and respond to social cues that are intuitive to human drivers [3,4,5]. In scenarios where AVs lack the ability to predict human driver behaviour, they use conservative behaviours to ensure safe operation [6] which can lead to traffic congestion and reduce the ability of other road users to infer an AV’s future actions [3,5,7].

Intersections are one of the most common locations for collisions between AVs and human-driven vehicles [8,9]. A 2017 report on AV collisions in California between 2014–2017 found that 23% of AV collisions occurred at an intersection [8,9]. A common collision orientation at intersections involved the AV being rear-ended by a conventional vehicle, often in response to conservative driving behaviour that was incongruent with the driving strategy of a typical human driver [8,9]. In particular, intersection navigation strategies change depending on the road orientation, type of intersection control device, and the behaviour of other road users [10,11]. For example, the decision around when to cross through an intersection is driven largely by the priority rules which are established by the control mechanism [10]. In a signalized intersection, priority rules are guided by the colour of the traffic light: go on green, stop on red. Priority becomes less clear in the dilemma zone, defined as the time before entering the intersection during a yellow light where drivers need to decide whether to stop or to go through [12,13]. For AVs waiting to make a left turn at a signalized intersection, correctly predicting whether a through driver in the dilemma zone will stop at the stop bar or proceed through can prevent a collision. If an AV accepts a smaller gap with which to proceed into the intersection, they may cause a side impact collision with the through driver. However, conservative behaviour where the AV chooses to wait at the yellow light, may result in a rear-end collision with a human driver expecting the AV to proceed. Similarly, conservative driving behaviour by AVs at sign-controlled intersections can lead to discomfort and reduced acceptance of AVs [14]. For example, in an on-road study, drivers reported a preference for AVs that combine a defensive driving style with confident priority-taking behaviour in complex scenarios like intersection navigation [14]. To reduce collisions with human-operated vehicles as well as the negative impacts of conservative AV behaviour during the mixed-autonomy transition period, AVs must be able to reliably model human driving behaviour [5,7].

Modelling the behaviour of other road users starts with perception. The perception stack of an AV includes multiple sensors, particularly a variety of cameras, in addition to Light Detection and Ranging (LiDAR) and RADAR systems [15,16]. Data collected by these sensors are fused and transformed to generate a real-time understanding of the AV’s environment, including the relative and absolute location, speed, and acceleration of other road users [16,17]. It follows that these variables represent the minimum amount of information available to an interacting AV. However, a proposed US mandate may soon make vehicle-to-vehicle (V2V) connectivity standard in all lightweight vehicles, including passenger cars [18], and Transport Canada has also established a plan to “coordinate and collaborate” with the US Department of Transportation to deploy V2V technology [19]. This advancement would dramatically increase the type of data available for use in behaviour prediction including information about the steering inputs, braking, and accelerator pedal status of other vehicles [20]. Therefore, in this study, behaviour of human-operated vehicles at intersections is predicted both with and without V2V data to compare the value added by the additional information to the task of predicting behaviour at intersections.

Various models have been previously proposed to improve intersection navigation. Dresner & Stone (2008) introduced a first-come, first-served model (FCFS) where AVs navigating an intersection requested a space and time to do so [21]. The first vehicle capable of occupying the space was granted priority by an intersection manager algorithm [21]. Adaptations for mixed autonomy were made to allow human drivers to continue to follow a typical traffic light system, while also granting permission to AVs to proceed through red lights in some scenarios. Simulations showed slight improvements in traffic efficiency at low AV saturations. However, differing priority rules for human drivers and AVs which conflict with current regulatory requirements would require substantial changes to road infrastructure [22]. Other models have enhanced the FCFS approach by introducing right-of-way-based models. In these systems, interacting vehicles bid based on their trajectories to pass through the intersection [23,24], but rely on all vehicles having V2V. 

There are fewer models that consider the individual behaviour of human drivers at intersections. One example is a reward function proposed by Sadigh et al. (2018) that rewarded an AV for influencing a human driver to take priority at a four-way stop [25]. Over time, the AV learned to reverse from the stop bar, demonstrating that an AVs behaviour can influence human driver behaviour (although the specific solution of reversing would not likely reduce collisions with, or be acceptable to, following drivers). Models of AV interactions with human drivers in other scenarios and with pedestrians have shown that incorporating human behaviour into AV decision-making can improve interactions by making AV behaviour more understandable to human road users, enhancing traffic efficiency, and reducing collision risk [3,5,26]. It follows that there is an opportunity to improve mixed autonomy interactions at intersections using a model informed by human factors.

Prediction models for non-intersection applications that account for human driver behaviour largely rely on opaque deep learning algorithms [27,28]. While these advanced approaches have considerably improved the ability of AVs to detect and avoid potential hazards, their decision-making is largely unexplainable [28]. A 2022 review of AV explainability found that for operations including planning, AV driving datasets lacked information about intermediate states, rendering the process of providing accurate explanations for AVs’ reasoning extremely challenging [29]. An inability to explain an AV’s reasoning creates issues around regulation, insurance, and law enforcement as well as trust imbalances with end-users [30]. Ultimately, miscalibrated trust may prevent the adoption of AVs or lead to misuse [29,31,32]. 

One way to provide explainability is to use inherently explainable models, which are transparent by design [29]. One such model class is decision trees, which are built by creating rules to partition training samples until only bins containing samples from a single class remain [33]. To avoid overfitting and improve robustness, typically decision trees are combined to create ensembles called forests [33]. Since the rules of each tree are explicit and follow a hierarchical logic, all intermediate states between the input data and output label are explainable. In this paper, results from one characteristically interpretable model (Time Series Forest) will be compared with results obtained from two state-of-the-art multivariate time series (MVTS) classifiers (ROCKET and HIVE-COTE 2.0). 

Additionally, decision tree-based classifiers provide feature importance, a measure of the extent to which a feature or variable influences the classification decision. Feature importances also provide information to assist in interpreting model reasoning [34]. Access to feature importance can provide insight into decision-making, make it easier to identify risks, and can help demonstrate compliance with regulations that require transparency [35,36]. End users are more likely to adopt and trust models when predictions are based on features that align with their expectations [37]. Feature importance acts as a bridge between model internals and user understanding. In this study, feature importances are calculated across the time series using a temporal feature importance method developed by Deng et al. [38].

To summarize, the purpose of this paper is three-fold. First, we compared the performances of an explainable MVTS classifier (Time Series Forest) with two less explainable, but state-of-the-art models (ROCKET and HIVE-COTE 2.0). This comparison was made using two datasets of human-driver priority-taking responses to hazardous, left turn scenarios at controlled intersections. Second, we additionally provided temporal feature importances for all features included in the models. Lastly, we compared performance of models that include and exclude data that may soon be made available by the implementation of V2V communication. Overall, AVs that can predict the intent of human drivers will be better able to model confident, rather than conservative behaviour in complex scenarios like intersection navigation. One way to improve these predictions is to monitor the most important features and include them as part of an AV’s behaviour planning model. Using explainable models to accomplish this task will enhance trust in AV-human driver interactions during the critical mixed-autonomy transition period.

## 2. Materials and Methods

### 2.1. Simulation Platform

**Figure 1:**
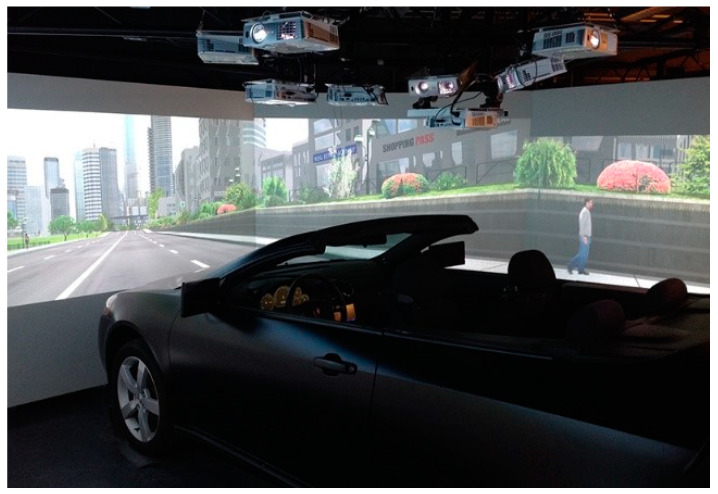
Fixed-base, full car driving simulator.

Data for this analysis were collected using a full vehicle driving simulator (Figure 1). The platform consists of an Oktal (Oktal Sydac, Toulouse, France) driving simulator, operating on SCANeR Studio v1.6 simulation software. To provide an immersive experience for participants, the driving environment was displayed using eight high fidelity projectors and 300° of wrap-around screens. The vehicle included a force-feedback enabled steering wheel and low frequency transducers installed under the driver’s seat.

### 2.2. Intersection Scenarios

**Figure 2:**
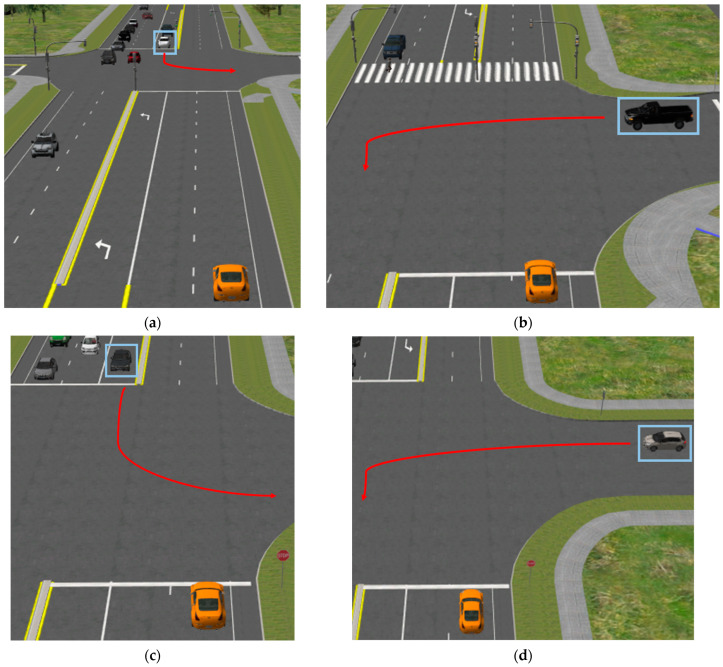
Left-turning vehicle hazards at intersections. Hazards are boxed in blue and participant drivers are in the orange vehicle. (**a**) Hazard vehicle turns from main road at signalized intersection; (**b**) hazard vehicle turns from minor road at half-signalized intersection; (**c**) hazard vehicle turns from major road at sign-controlled intersection; (**d**) hazard vehicle turns left from minor road at sign-controlled intersection.

Participants encountered the left-turning hazard vehicles at intersections with two different control infrastructures, sign-controlled and signalized. Two of the four intersections were controlled by a four-way stop such that both the participant and the left-turning hazard vehicle were facing a stop-sign upon approach (Figure 2c,d). Of the two signalized intersections, one was fully controlled by traffic signals (i.e., signalized), but did not include countdown timers on the pedestrian signals (Figure 2a). The final intersection was half-signalized, with signalized traffic on the major road, and stop-sign-controlled traffic on the intersecting minor road (Figure 2b). This configuration includes a pedestrian crossing on the far side to provide a reasonable scenario where the participant driver was facing a yellow signal, and the hazard vehicle could make a left turn into the path of the participant driver. These four scenarios were designed to obfuscate the decision to take priority or to wait at the stop bar. A similar likelihood of each decision was confirmed by pilot testing.

The participant driver was instructed to travel the major road for the duration of the experiment. This road was four lanes wide (two lanes in each direction), with a fifth left turning lane at the signalized intersections. The speed limit was 60 km/h along the entire stretch of roadway. The minor roads, which intersected the major road at all intersections, were two lanes wide (one lane in each direction) and were not separated by a median. 

The four left turning hazards included two which turned left into the participant’s path from the major road (Figure 2a,c), and two which turned left into the participant’s path from the minor roads (Figure 2b,d). Combined with the signalized and sign-controlled intersection types, this created four, distinct left-turning hazards. 

At the signalized intersections, as the participant vehicle came within 54–59 m of stop bar, the light would change from green to yellow. This location was well within the dilemma zone as defined by a 2014 review [13], and was optimized during pilot testing to ensure ambiguity. Once the light turned yellow, the left turning vehicle would begin to accelerate from stopped at a rate of 0.98 m·s^−2^. At the sign-controlled intersections, as the participant began to slow for the stop sign, so did the hazard vehicles. However, rather than coming to a full stop, the hazards then rolled the stop and proceeded into the intersection at a rate of 2.0 m·s^−2^.

### 2.3. Participants and Procedures

To obtain the dataset, 125 licensed drivers (79 women, 45 men, 1 non-binary) responded to all four hazardous scenarios. Participants were between 17 and 65 years old, and had a mean age of 23 years. All participant drivers held an Ontario G2, G, or out-of-province equivalent license which permitted them to drive independently. Study procedures were approved by the Research Ethics Board and all drivers provided informed consent prior to participation. Drivers were screened for risk factors of simulator adaptation syndrome before participation [39]. Participants completed a 5-min practice drive to familiarize themselves with the vehicle controls and scenario. The practice drive included the same intersections used in the experimental drive, without requiring priority decisions or emergency responses to the hazard vehicles. Prior to the experimental drive, drivers were told to observe the speed limit and to remain in the right lane but were not made aware of the hazards. The four hazardous intersection scenarios were presented in a counterbalanced order to mitigate learning effects. 

### 2.4. Dataset Construction and Preprocessing

**Figure 3:**
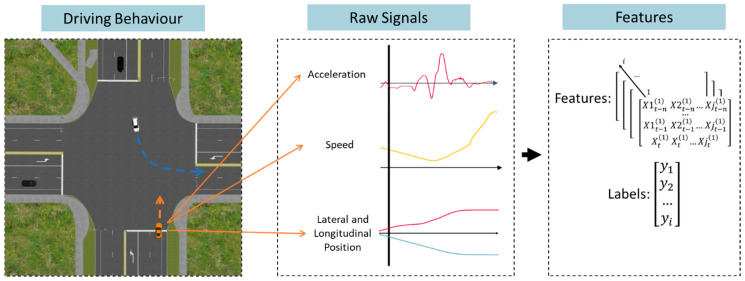
An overview of the conversion process from driving behaviour to a 3d matrix of features which can be used to train a multivariate time series classifier. Signals were collected at 100 Hz from an instrumented full vehicle driving simulator. Signals included acceleration, speed and position of both the participant’s vehicle (orange, as pictured) and the turning AV (white), as well as brake pedal pressure, gas pedal pressure, and steering wheel angle. Additional features were created by combining or transforming these signals. Segments were selected to include 7.5 s of driving preceding hazard onset. Lastly, the segments were used to construct a 3d feature matrix with axes for time, hazard instance, and feature. The binary labels, either stop at the stop bar (“no go”) or proceed through the intersection (“go”) were recorded based on the priority-taking behaviour of the participant driver.

Dataset construction began by recording driving signals generated during the interaction of participant drivers with AVs during four LTAP scenarios. The simulator acquisition computer sampled each approach and hazard response at a rate of 100 Hz. Variables of interest, including lateral and longitudinal speed, acceleration, and position were exported from the simulation software (SCANeR Studio v1.6). Scenarios where the hazard vehicle was occluded by auxiliary traffic or otherwise did not trigger correctly were removed from the analysis. The remaining post-processing was completed in Python (v3.10.10). For each scenario, 7.5 s of driving preceding hazard onset were included in the dataset. Hazard onset was defined as the first lateral movement of the hazard vehicle into the intersection. This included all the precursor information a driver would collect to make a priority-taking decision. Multivariate time series data were stored using a 3d array format with axes for time, hazard instance, and feature. Figure 3 provides an outline of the segment extraction and feature engineering process. 

**Figure 4:**
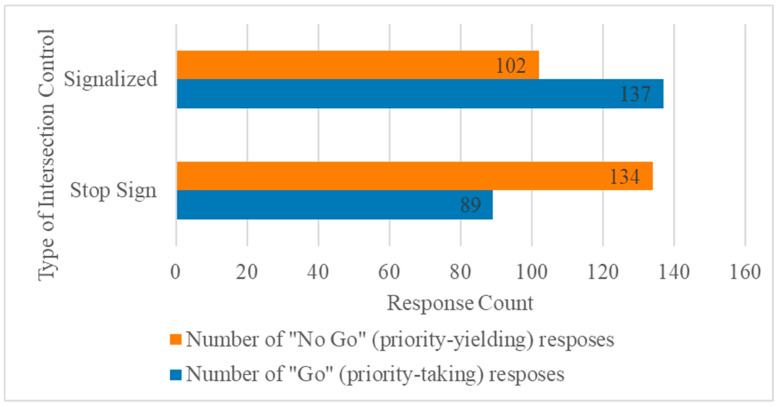
Binary class membership distribution (go vs. no-go) for signalized and stop-sign controlled left turn scenarios.

Both signals which could be reasonably obtained by perception-stack sensors onboard an AV and signals which can be obtained from V2V communications for the oncoming vehicle, such as pedal positions and steering wheel angle [20], were included in the analyses. A full list of the time series included in the dataset are outlined in Table 1. Priority-taking behaviour (the categorization target) was split between the datasets for signalized and sign controlled intersections (Figure 4).

### 2.5. Feature Selection

Features were selected for inclusion in the model using a correlation-based feature subset selection procedure called Merit Score for Time-Series (MSTS) [40]. This multivariate time series specific procedure was selected because it enables dimensionality reduction while preserving the inter- and intra-time series relationships. Additionally, using MSTS eliminates the need for feature engineering or aggregation, which would complicate real-time application since the AV would not have access to all features until the end of the scenario. Use of MSTS also preserves the interpretability of the features. 

Correlations between features $\left(Yff\right)$ and between features and labels (Ycf) were calculated using the Adjusted Mutual Information Score [41]. A merit score was then calculated for each feature subset using the following equation:

(1) $$MSTS=\frac{n\bar{Y_{cf}}}{\sqrt{n+n\left(n-1\right)\bar{Y_{ff}}}}.$$

Given that n is the number of features in the subset, merit scores are bounded between 0 and 1, with higher scores representing feature sets which have higher classification accuracy and lower correlation between features within the set. 

First, all possible combinations of two features were evaluated to find the pair with the highest MSTS score. Then, additional features were added one at a time to the highest scoring feature subset until the merit score no longer improved. 

### 2.6. Model Training and Sktime Classifiers

After the final set of features was determined, training and testing sets were generated by combining the features with their labels (i.e., the driver’s priority decisions at each intersection). A k-fold cross validation procedure (k = 5) was used to create training and testing groups. Each fold included an 80/20 ratio of training and testing data. The classification metrics for the final model were averaged across folds.

Performance was compared between three time series classification models (ROCKET, HIVE-COTE 2.0, and Time Series Forest) from the Sktime library [42]. Sktime is a python library designed specifically for machine learning with time series and includes support for many state-of-the-art models, including ROCKET and HIVE-COTE 2.0, but also provides feature importance calculation functionality for explainable models like Time Series Forest. 

ROCKET uses random convolutional kernels to transform time series into features which are then used to train a linear classifier [43]. To avoid computational expense, a very large amount and variety of random kernels are used instead of implementing multiple convolutional layers with learned weights. ROCKET has gained popularity for its ability to efficiently handle large datasets and achieve competitive performance with CNNs. 

Hierarchical VotE Collective of Transformation-based Ensembles (HIVE-COTE 2.0) is a meta-ensemble of classifiers [44]. It combines four techniques including a shapelet-based classifier, an ensemble of ROCKET classifiers, the Temporal Dictionary Ensemble, and the interval based Diverse Representation Canonical Interval Forest Classifier. HIVE-COTE 2.0 works by training each model independently and producing a probability of membership for each class. Then, the four classifiers are combined using a weighted estimate, calculated using individual performance metrics.

Time Series Forest (TSF) is a tree-ensemble method proposed by Deng et al. (2013). TSF randomly samples intervals of each time series and calculates the mean, standard deviation, and slope for each sample interval to create an interval feature [38]. Entrance gain, a combination of entropy gain and margin, is used as the splitting criterion for the nodes. Entropy gain is a common criterion for tree-based classifiers and quantifies how much information a feature provides about a class. Margin is the minimum distance between a split threshold candidate and the nearest feature value. Like other decision tree ensembles, TSF is a collection of trees built top-down. The forest predicts class membership by majority vote from all the trees. 

### 2.7. Temporal Importance Curve

Temporal importance curves capture the contribution of each time point to the construction of the decision trees. Specifically, the temporal importance plots denote total entropy gain for each time point. If a time point was included in intervals with higher overall entropy gain, then it ranks higher on the plot. Curves for each calculated feature (mean, standard deviation, and slope) are plotted separately. Since randomly sampled time intervals are more likely to contain time points in the middle of the segment, temporal importance is biased towards the center of the plot.

## 3. Results and Discussion

In this paper, one inherently explainable and two state-of-the-art time series classifiers were trained to model a binary classification task for four datasets. All included human drivers’ “go” or “no-go” responses to an ambiguous left-hand turn scenario. Two datasets included instances at signalized intersections, while the other two datasets included instances at a stop-sign-controlled intersection. Within the signalized and stop-sign controlled groups, one dataset included only variables that could be captured by the sensors on an AV while the other also included variables which could be transmitted by V2V communication [19] (Table 1). 

### 3.1. Feature Selection

After applying the described feature selection process for models involving both control devices (stop-sign and signalized) and feature sets (AV sensors only and with V2V variables), the selected features are presented in Table 2. The final models were trained using only these features.

**Figure 5:**
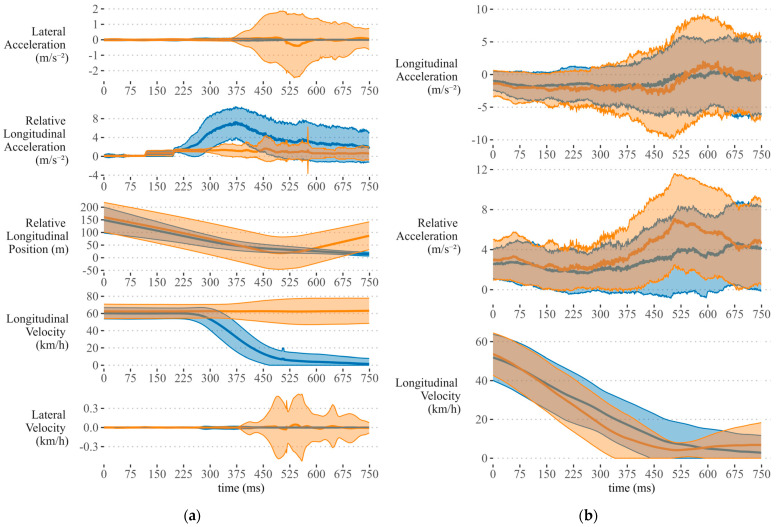

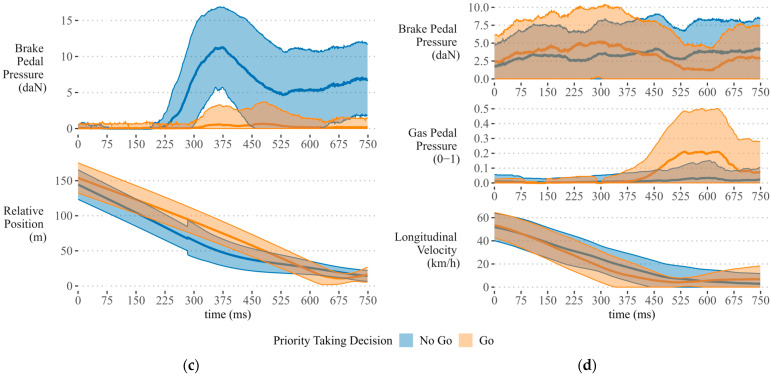
Plots of selected features for each control type/dataset combination averaged across participants and separated by priority-taking decision. Shaded regions represent the standard deviation around the mean: (**a**) Selected features from the signalized intersection data; (**b**) selected features from the stop-sign-controlled data; (**c**) selected features from the signalized intersection data including the V2V variables; (**d**) selected features from the stop-sign-controlled data including the V2V variables.

Figure 5 shows the time series curves of the selected features averaged across the participants for each time point. For each control type/dataset combination, the time series is plotted with standard deviation shaded around the mean. Additionally, the participants are separated by priority-taking decision (i.e., the binary classification target) over the sampled interval. 

### 3.2. Model Performance

Results comparing TSF to the ROCKET and HIVE-COTE 2.0 classifiers are shown in Table 3. In terms of accuracy, TSF performed similarly to ROCKET and HIVE-COTE 2.0, on the stop-sign-controlled datasets, but slightly worse than ROCKET and HIVE-COTE 2.0 on the signalized data. Within the classifiers, there was no consistent performance difference between the sensor only and V2V models. Overall, all models performed significantly better on the signalized dataset than on the stop-sign-controlled dataset. 

Looking closer at the signalized datasets, the TSF models maintain equivalent, if not greater prediction accuracy when compared to similar driver behaviour prediction models [45,46]. In a 2021 study, researchers trained three classifiers to predict drivers’ decisions to stop or go while in the dilemma zone at a signalized intersection [45]. These models included a linear SVM, a polynomial SVM, and an ANN. Similarly, a 2016 investigation used a gradient-boosting logit model to predict drivers’ stop or go behaviour when faced with a yellow light [46]. Classification accuracy for all models are compared to results from the current study in Table 4. 

TSF did not perform as accurately on the stop-sign-controlled datasets when compared to similar, existing models. For example, a logistic regression model designed to predict crashes at stop-sign-controlled intersections [42] (Table 5) outperformed all three models evaluated in this paper. This may be due to the use of a more complex derived feature or the more extreme differences in the outcome behaviours. Given the relative subtlety of a “go” decision when compared to a collision, it is possible that this model may not generalize well to the dataset used in this paper.

Also included in Table 3 are the true positive rate (TPR) and true negative rate (TNR) for each model. TPR is the rate of responses correctly predicted to be “no go” responses, while TNR is the rate of responses correctly predicted to be “go”. The inverse of these measures, false negative rate (FNR) and false positive rate (FPR), can be calculated by subtracting TPR and TNR from 1, respectively. FNR is the rate of “no go” responses predicted to be “go” responses and FPR is the rate of “go” responses predicted to be “no go” responses.

TPR was greater than TNR across all models. In other words, the models were better able to correctly predict “no go” responses than “go” responses. The ability to correctly predict human drivers’ yielding behaviour provides an opportunity for Avs to take priority more often at sign-controlled intersections when they may have otherwise waited for traffic to clear before proceeding [48]. This can reduce the likelihood of rear-end collisions with human drivers who expect more aggressive, priority-taking behaviour [6,48].

For the stop-sign-controlled classification task, TSF was slightly more likely than the other two models to misclassify “no go” responses when relying solely on AV sensor data. However, interestingly, this trend is reversed for the sign-controlled data with the V2V variables. One explanation for the reduction in false positives given the selection of brake and gas pedal pressure in this feature set (over other measures of longitudinal control) is that foot pedal activation can help to differentiate between drivers with otherwise similar speeds or deceleration rates. In other words, speed change due to braking is a more direct indicator of a driver’s intention to stop than speed change due to rolling resistance or slope change. This is well demonstrated by a 2021 experiment which attempted to cluster driver approach behaviour at stop signs using speed measurements alone [10]. Most observations (86%) were assigned to a single cluster which included trajectories with little noticeable speed change but a variety of priority-taking behaviours [10]. In this case, knowing the status of the brake or gas pedal would likely help to differentiate between the variety of behaviours represented by this cluster, since pedal application is a direct indicator of the driver’s intention to stop or go. 

Also at the stop-sign-controlled intersections, the addition of V2V features into the dataset led to substantial increases in TPR of all models. More modest increases in accuracy and true negative rate (TNR) were also observed. For the TSF model, the addition of V2V features led to a 14% increase in TPR and a 10% increase in TNR. Thus, the model was able to more accurately predict both “go” and “no go” responses when compared to the TSF model which did not include V2V features. This increase in TNR and TPR may lead to decreased incidence rate of both rear-end and side impact collisions between human drivers and the AV. 

### 3.3. Temporal Importance Curves

Temporal importance curves were plotted for each feature remaining after completing the feature selection protocol. Each feature calculated by TSF was plotted separately. Each plot included the mean, standard deviation, and slope of each randomly sampled interval. The feature importance is the sum of the entropy gain for all the nodes associated with a time point for each feature [49].

**Figure 6:**
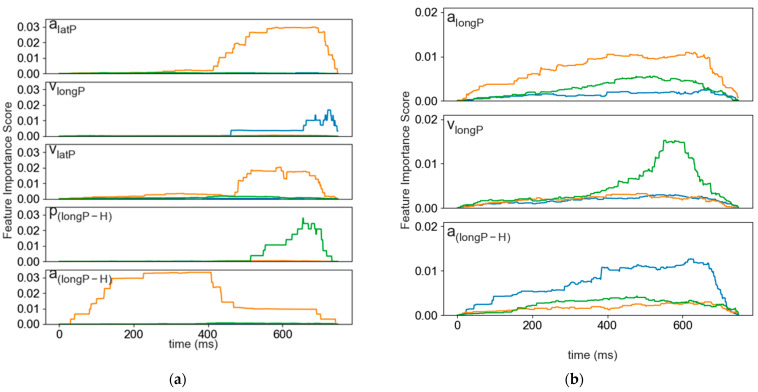

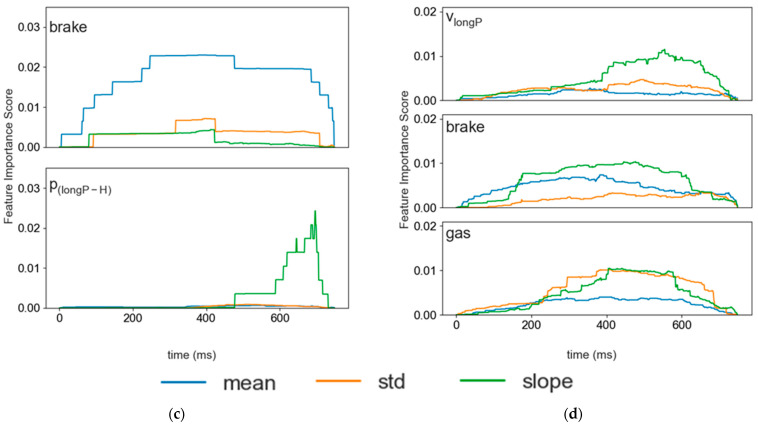
Temporal importance curves for mean, standard deviation (std), and slope of selected features. Calculated using entropy gain. (**a**) Signalized intersection dataset, which included participant lateral acceleration ($a_{latP})$, participant longitudinal velocity ($v_{longP}$), relative longitudinal acceleration difference between the participant and hazard ($a_{longP-H}$), participant lateral velocity ($v_{lat1}$), and relative difference in longitudinal position between the participant and hazard ($p_{longP-H}$); (**b**) stop-sign-controlled dataset, which included participant longitudinal acceleration ($a_{longP})$, participant longitudinal velocity ($v_{longP}$), relative longitudinal acceleration difference between the participant and hazard ($a_{longP-H}$); (**c**) signalized intersection dataset when the V2V variables were included. This included participant brake pedal pressure (brake) and relative difference in longitudinal position between the participant and hazard ($p_{longP-H}$); (**d**) stop-sign-controlled dataset when the V2V variables were included. These features were participant longitudinal velocity ($v_{longP}$), participant brake pedal pressure (brake), and participant gas pedal pressure (gas).

For the signalized, sensor-only dataset, all but one of the selected features demonstrated a peak in feature importance scores between 0.5–2.5 s prior to stop bar incursion (Figure 6a). The remaining feature, the relative longitudinal acceleration ($a_{longP-H}$) peaked much earlier in the 3–5.5 s prior to stop bar incursion. This feature also had the highest feature importance values of all the selected features. Similarly, the mean of the brake pedal pressure feature from the V2V inclusive dataset reached its maximum feature importance scores starting about 5.25 s prior to stop bar incursion (Figure 6c). In other words, both signalized datasets had at least one feature that demonstrated a high feature importance score early in the time series. It follows that the advantage of including the V2V data is that fewer features are required to achieve slightly greater accuracy and still provide an early indicator of future priority-taking behaviour.

The temporal importance curves for the sign-controlled data do not provide as much information about the importance of the selected features. Of the three features selected, participant longitudinal speed contributes most to the model regardless of the inclusion of the V2V variables. In Figure 6a,b, there is a peak in the slope feature for participant speed. This peak captures a difference in slope between the two classes in the interval 1.5–2.5 s prior to the end of the segment. The other temporal importance curves for these models do not illustrate an obvious difference between the go and no-go classes. Interestingly, there is some evidence in Figure 5 that gas pedal pressure may provide some additional variability on which to separate the priority-taking responses, with “go” drivers providing more input in the 2.5 s before stop bar incursion. Further investigation is required to understand why this difference is not being reflected in the temporal importance curve for the sign-controlled scenario. 

## 4. Limitations

The study has some limitations to consider. Firstly, the observed priority-taking at stop-sign intersections did not fully reflect the aggressive behaviour observed in naturalistic studies. Participant drivers were much less likely to roll or run the stop sign when compared to behaviour in similar on-road studies [11,50]. In addition, while binary labels using “go” and “no-go” groups worked well for signalized intersections, this classification was less clear for the stop-sign-controlled scenarios. A similar, previous study of priority-taking at stop signs clustered driver behaviour into five groups [11] which may indicate that behaviour at stop signs is more varied or driver-specific than behaviour at light-controlled intersections. Looking at which occurrences were misclassified, minor intrusions into the intersection were more likely to be classified as no-go than any other behaviour. Minor intrusions may not always represent priority-taking; rather, human drivers and AVs have both used this strategy to signal an intention to proceed [7,51].

Evidence from existing simulator studies shows that repeated hazard exposure primes human drivers to expect future hazards, thereby decreasing perception and response times [50,51,52]. Given that this study was designed to garner genuine human driver responses to hazardous scenarios, this experiment was purposefully limited to the four LTAP scenarios discussed. While this does limit the size of the dataset and the generalizability of the models, future work should extend the proof of concept provided in this study to include more drivers and other hazardous scenarios. 

## 5. Conclusions

The experimental results from this study show that explainable methods like TSF can be applied to multivariate time series classification problems with similar accuracy to state-of-the-art classifiers like ROCKET or HIVE-COTE 2.0. Additionally, analysis of the temporal importance curves showed that for signalized intersections, a human operator’s longitudinal control of the vehicle can be used to accurately predict priority-taking behaviour in a dilemma zone, and these predictions are possible as early as 5.5 s before stop bar incursion. Given the opportunity to use data transmitted by V2V communication systems, some of these variables could be replaced by the inclusion of brake pedal pressure, thereby reducing the complexity of the model and reducing the computation time without affecting accuracy. 

Predicting the priority-taking behaviour of drivers at stop-sign-controlled intersections was less successful, but the inclusion of V2V variables did improve both the true negative and true positive rate of the classifier. Specifically, the inclusion of brake and gas pedal pressure improved the ability of the TSF model to predict the decision to “go”. Since drivers do not have such large variations in speed during priority-taking at stop-sign-controlled intersections when compared to light-controlled intersections, it follows that, with some additional feature engineering, gas pedal application could be a more predictive measure of longitudinal control than speed or acceleration. 

The next steps for this research include applying the selected features, at the time intervals corresponding to their peak feature importances, to an AV planning model. Such a model should enable AVs to predict human drivers’ behaviour earlier and more accurately, allowing for reduced reliance on conservative driving behaviours to successfully navigate hazardous scenarios like the left-turn scenario used in this study. Additionally, this work demonstrates that inherently explainable models, which are necessary to address trust imbalance [32] and alleviate issues related to regulation [29], are competitive with other state-of-the-art time series classifiers. Finally, the analysis of the sign-controlled data indicates that the inclusion of V2V variables improves the ability of the models to identify the hazardous “go” priority-taking responses, thereby minimizing risk of a collision with the through driver without relying exclusively on conservative driving behaviours.

## Figures and Tables

**Table 1:** Names and descriptions of time series features extracted from the simulator. V2V features are indicated in the description. Feature subscripts refer to direction, either longitudinal (long) or lateral (lat) and which vehicle was measured, one or both of participant (P) or hazard (H).

Feature	Description and Units
$v_{longP}$	The longitudinal velocity of the participant vehicle (m/s)
$v_{longH}$	The longitudinal velocity of the left-turning hazard vehicle (m/s)
$v_{longP-H}$	The difference in longitudinal velocity between the participant and hazard vehicle (m/s)
$v_{latP}$	The lateral velocity of the participant vehicle (m/s)
$v_{latH}$	The lateral velocity of the left-turning hazard vehicle (m/s)
$v_{latP-H}$	The difference in lateral velocity between the participant and hazard vehicle (m/s)
$a_{longP}$	The longitudinal acceleration of the participant vehicle (m·s^−2^)
$a_{longH}$	The longitudinal acceleration of the left-turning hazard vehicle (m·s^−2^)
$a_{longP-H}$	The difference in longitudinal acceleration between the participant and hazard vehicle (m·s^−2^)
$a_{latP}$	The lateral acceleration of the participant vehicle (m·s^−2^)
$a_{latH}$	The lateral acceleration of the left-turning hazard vehicle (m·s^−2^)
$a_{latP-H}$	The difference in lateral acceleration between the participant and hazard vehicle (m·s^−2^)
$p_{longP}$	Longitudinal position of the participant vehicle with respect to the global coordinate system of the simulation (m)
$p_{longH}$	Longitudinal position of the left-turning hazard vehicle with respect to the global coordinate system of the simulation (m)
$p_{longH-P}$	The difference in longitudinal position between the participant and hazard vehicle (m)
$p_{latP}$	Lateral location of the participant vehicle w.r.t the global coordinate system of the simulation
$p_{latH}$	Lateral location of the left-turning hazard vehicle w.r.t the global coordinate system of the simulation (m)
$p_{latP-H}$	The difference in lateral position between the participant and hazard vehicle (m)
$d_{gap}$	Distance between the center of mass of the participant vehicle and the location of the lane marker (m)
$j_{longP}$	The rate of change of longitudinal acceleration of the participant vehicle (m·s^−3^)
brake	V2V variable: Pressure exerted on the brake pedal (daN)
gas	V2V variable: Pressure exerted on the accelerator
steer	V2V variable: Steering wheel angle (°)

**Table 2:** Features selected for inclusion in the final classifiers, by intersection control type and feature set.

Control Type/Dataset	Feature Set	Features Included in Final Model	Final MSTS Score
Signalized	AV Sensors Only	$a_{latP}$ $,\;v_{longP}$ $,\;v_{latP}$ $,\;p_{longP-H},\;a_{longP-H}$	0.881
Stop-Sign Controlled	AV Sensors Only	$a_{longP}$ $,\;v_{longP}$ $,\;a_{longP-H}$	0.272
Signalized	with V2V variables	brake $,\;p_{longP-H}$	0.876
Stop-Sign Controlled	with V2V variables	$v_{longP}$ , brake , gas	0.280

**Table 3:** Comparison of performance metrics for three time series classifiers.

Model Name	Control Type	Dataset	Accuracy	AUC ROC	True Positive Rate (TPR)	True Negative Rate (TNR)	f1-Score
Time Series Forest	Signalized	Sensors Only	0.94	0.91	0.99	0.91	0.92
Time Series Forest	Signalized	V2V	0.93	0.93	0.96	0.91	0.93
Time Series Forest	Stop-sign-controlled	Sensors Only	0.72	0.75	0.76	0.58	0.81
Time Series Forest	Stop-sign-controlled	V2V	0.77	0.75	0.87	0.64	0.81
ROCKET	Signalized	Sensors Only	0.97	0.98	0.98	0.96	0.96
ROCKET	Signalized	V2V	0.96	0.97	0.98	0.95	0.95
ROCKET	Stop-sign-controlled	Sensors Only	0.72	0.73	0.79	0.62	0.77
ROCKET	Stop-sign-controlled	V2V	0.79	0.79	0.83	0.73	0.83
HIVE-COTE 2.0	Signalized	Sensors Only	0.96	0.97	0.98	0.95	0.96
HIVE-COTE 2.0	Signalized	V2V	0.96	0.97	0.98	0.95	0.95
HIVE-COTE 2.0	Stop-sign-controlled	Sensors Only	0.71	0.70	0.80	0.57	0.77
HIVE-COTE 2.0	Stop-sign-controlled	V2V	0.78	0.76	0.87	0.65	0.83

**Table 4:** Comparison of model accuracy with previous literature findings that also include stop bar incursions at signalized intersections.

Model	Accuracy	Interpretable?	Features Used?
Linear SVM [47]	0.82	N	Vehicle speed, vehicle location relative to stop bar, time of day
Polynomial SVM [47]	0.89	N	
Artificial Neural Network [47]	0.91	N	
Statistical Logit [46]	0.72	Y	Signal timing information, occupancy time, time gaps, adjacent lane, preceding vehicle’s decision
Boosting Logit [46]	0.91	Y	
Time Series Forest	0.91	Y	Lateral acceleration of participant vehicle, difference in lateral acceleration of the participant and hazard vehicles, longitudinal and lateral velocities of participant vehicle, difference in longitudinal position between the participant and hazard vehicles
ROCKET	0.97	N	
HIVE-COTE 2.0	0.97	N	
Time Series Forest	0.93	Y	Brake pedal force, difference in longitudinal position between the participant and hazard vehicles
ROCKET	0.97	N	
HIVE-COTE 2.0	0.97	N	

**Table 5:** Comparison of results with models reported in the literature that also predict stop bar incursions at sign-controlled intersections.

Model	Accuracy	Interpretable?	Features Used?
Early [47]	0.95	Y	Required deceleration parameter
Intermediate [47]	0.98	Y	average deceleration magnitude required to stop the vehicle given some velocity and stopping distance, brake application
Delayed [47]	0.98	Y	
Time Series Forest	0.72	Y	difference in longitudinal acceleration of the participant and hazard vehicles, longitudinal acceleration of the participant vehicle, longitudinal velocity of the participant vehicle
ROCKET	0.71	N	
HIVE-COTE 2.0	0.72	N	
Time Series Forest	0.76	Y	Brake pedal force, gas pedal force, longitudinal velocity of participant vehicle
ROCKET	0.79	N	
HIVE-COTE 2.0	0.78	N	

## Data Availability

The driving data exported from the simulator are available at https://github.com/eziraldo/DRiVELab-BnE2021 (accessed on 13 June 2024).
